# RNA Relics and Origin of Life

**DOI:** 10.3390/ijms10083420

**Published:** 2009-07-31

**Authors:** Jacques Demongeot, Nicolas Glade, Andrés Moreira, Laurent Vial

**Affiliations:** 1 University Joseph Fourier of Grenoble, TIMC-IMAG UMR UJF/CNRS 5525, Faculty of Medicine, 38700 La Tronche, France; E-Mail: Nicolas.Glade@imag.fr (N.G.); 2 Departamento de Informática, Universidad Federico Santa María, Casilla 110-V, Valparaíso, Chile; E-Mail: amoreira@inf.utfsm.cl (A.M.); 3 Département de Chimie Moléculaire, UMR CNRS 5250, Université Joseph Fourier, 301 rue de la chimie, BP 53, 38041 Grenoble Cedex 9, France; E-Mail: Laurent.Vial@ujf-grenoble.fr (L.V.)

**Keywords:** RNA relics, tRNA loops, micro-RNA, viral RNA, archetypal genome, co-evolution

## Abstract

A number of small RNA sequences, located in different non-coding sequences and highly preserved across the tree of life, have been suggested to be molecular fossils, of ancient (and possibly primordial) origin. On the other hand, recent years have revealed the existence of ubiquitous roles for small RNA sequences in modern organisms, in functions ranging from cell regulation to antiviral activity. We propose that a single thread can be followed from the beginning of life in RNA structures selected only for stability reasons through the RNA relics and up to the current coevolution of RNA sequences; such an understanding would shed light both on the history and on the present development of the RNA machinery and interactions. After presenting the evidence (by comparing their sequences) that points toward a common thread, we discuss a scenario of genome coevolution (with emphasis on viral infectious processes) and finally propose a plan for the reevaluation of the stereochemical theory of the genetic code; we claim that it may still be relevant, and not only for understanding the origin of life, but also for a comprehensive picture of regulation in present-day cells.

## 1. Introduction

It has become customary to refer as “RNA relics” or “molecular fossils” to the several kinds of RNA (or more generally XNAs with X for the D of DNA, the R of RNA, or the P of PNA, the peptide nucleotidic acids potential precursors of the RNAs in the primordial forms of life) sequences that perform highly preserved functions and appear almost everywhere in the tree of life. Numerous nucleic acid sequences present in genomes (like viral genomes) or products of genomes such as small RNAs or transfer RNAs (tRNAs) loops are often thought to be RNA relics since they are common to many different species and because they share high interspecific invariant parts. In the present paper, we propose a scenario of co-evolution of all these genomes and also suggest that ancestral processes of genetic complementarity or of genetic information encoding (the hypothesis of stereochemical links between nucleic and amino acids furnishing a plausible explanation for the birth of the genetic code) are still present and active in present cells playing also new roles, particularly in cell regulation and in immunity.

Small RNAs are sequences of 10 to 30 bases, of which several types have been identified–the micro-RNAs (or miRs, whose mean length in human is 22 bases), the small nuclear RNAs (or snRNAs) and their subclass the numerous small nucleolar RNAs, parts of the UnTRanslated (UTR) genomes, and the small interfering RNAs (siRNAs) coming from the degradation of RNAs, notably cytoplasmic messenger RNAs (mRNAs).

MicroRNAs have been found in all living realms (animals, plants, bacteria and viruses) with strong interspecific sequence homologies (miRs are distributed in several large phylogenetic families called miR-*x*, with *x* being the number of the family, within which the parts related to the regulation of their expression vary but where the mature sequences conserve strong homologies [1,2]), suggesting they derive from common ancestors and they probably fulfil vital functions, either shared by these species, or related to a strong co-evolutionary coupling, *e.g.,* involving transversal carriers of genetic material like viruses or phages or related to ancestral mechanisms of immune defence.

By base-pairing with mRNAs, miRs (and more generally small RNAs) inhibit their related functions (*e.g.,* translation for mRNAs) during a lapse of time, depending on the force of hybridization. This is what is called the ‘silencing’ process. Moreover, miRs can cause the degradation of their targets when coupled to specific protein complexes (called RISC for RNA-Induced Silencing Complex) that contain an RNase. Small RNAs with important regulatory roles are not limited to eukaryotes. They have been found recently in *Escherichia coli* and in other bacteria. As in eukaryotic cells, these small RNAs act by base-pairing with target mRNAs, resulting in changes in the translation and stability of these mRNAs. Finally, miRs themselves can be down-regulated, increasing the complexity of the regulatory loops in which the are implied. They can either be directly silenced by other miRs (miR duplexes) [3] or their transcription can be regulated by factors like p53 [4].

MicroRNAs are known to play an important role in morphogenesis (during the development of embryos or continuous cell differentiation in mature organisms), cell growth and death control, bacterial stress responses and virulence, and more generally global “gene” silencing since they are able to inactivate efficiently specific messages of hundred targets at the same time [5,6].

Increasing evidence also indicates that RNA interference, via miR silencing, may be used to provide antiviral immunity in mammalian cells [7]: Human miRs inhibit the replication of a primate virus, whereas a virally-encoded miRNA from HIV inhibits its own replication [8]. Moreover many viral-encoded miRs have been discovered, mostly in viruses transcribed from DNA genomes (herpesviruses, polyomaviruses, and retroviruses) showing an influence on their biogenesis. Conversely, viral interactions with miRs of the host have been identified, like those of HCV with the human miR-122, which upregulates viral RNA levels [9–11].

In the following, archetypal genomes, both theroretical ones obtained by computing the sequence as solution of a combinatory variational problem (Section 2) and natural RNA relics (Section 3), are detailed. In Section 4, sequence alignments provide evidence of similarities between theoretical archetypal genomes, RNA relics and some viral genomes. They suggest the existence of common ancestors for small RNAs and tRNA loops, and a coevolution between small RNAs and viral genomes (Section 5). Finally, we end the article with Section 6 that presents how some biological processes (like immunity or genetic information encoding) could have been inherited by the existing living systems from very ancestral ones that emerge at the origins of life or in prebiotic conditions, and that imply small RNAs. Smaller constituents like amino-acids or other metabolites are susceptible to interact directly with single XNA codons. This stereochemical mechanism furnishes an understanding for a coupled emergence of the genetic–triplet–code and peptide synthesis.

## 2. An Archetypal Genome

**Figure 1.**
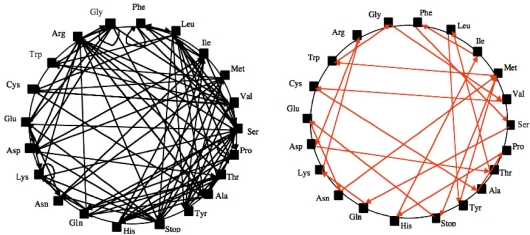
Search for a Hamiltonian circuit (it is a graph cycle through a graph that visits each node exactly once) on the graph of possible overlaps between synonymy classes of the genetic code (left) and a solution AB (right).

Plausible circular or hairpin-shaped XNA’s (called “Archetypal Base” AB or “Ancestral Loop” AL) have been found by solving a variational problem [12–13] consisting in finding the shortest RNA made of the succession (with overlaps) of triplets with one and only one representative among the synonymy classes of the genetic code (Figure 1).

**Figure 2.**
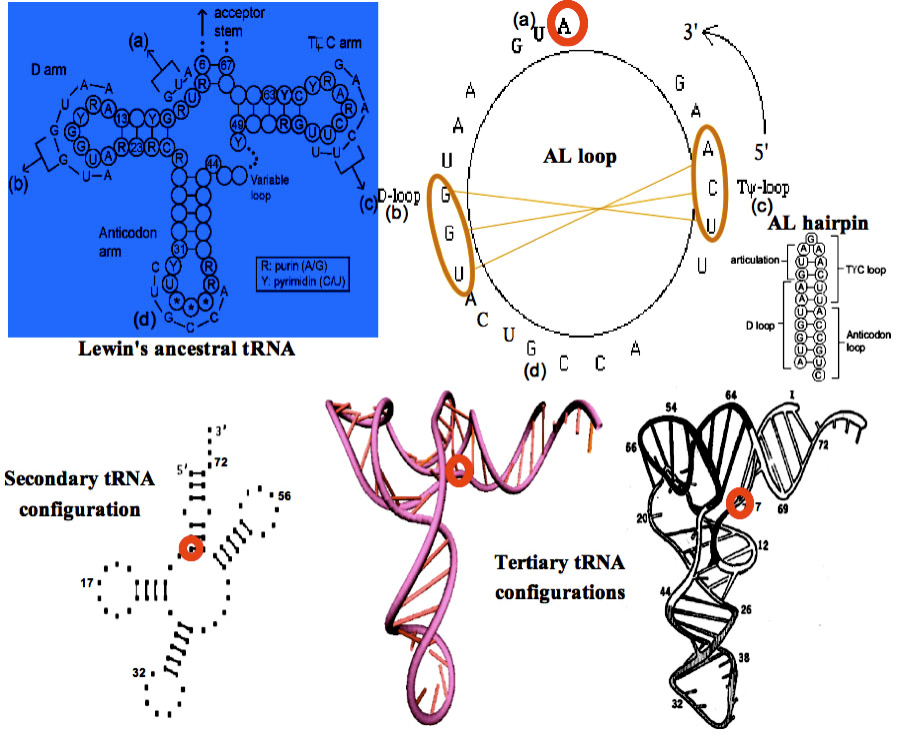
Secondary structure of the Lewin’s template tRNA with 4 parts (a,b,c,d) corresponding to the Gly-tRNA of *Arabidopsis thaliana* (top left); AL loop and hairpin (top right) with the pivot A and two complementary triplets (top right) causing the tRNA tertiary structure from secondary one (bottom).

The AL loop can be distinguished by using plausibility arguments [14–19] as baricentre of a selected subset of the 29,520 solutions (having the most stable hairpins as possible secondary structure) in the space of classes of equivalence of chains for circular permutations, for i) circular Hamming distance, ii) distance equal to 22 minus the length of the maximal common substring, and iii) minimal evolution shuffling similarity, *i.e.*, minimum number of consecutive deletions of maximal common substrings needed for obtaining the same final sequence. Thus characterized by its stability properties alone [18] (which includes a strong version of the stereochemical hypothesis of the genetic code), AL turns out to be also the closest sequence - for a “cut” distance, corresponding to minimal Hamming distance between four AL fragments and tRNA parts - to about 7,000 transfer RNAs (tRNA) taken from about 180 species found in the //felix.unife.it/Root/d-Biology/d-Genetics-and-evolution/d-tRNA-sequences/t-tRNA-compilation database. Reduced to sequences of conserved domains in primary structure, they are considered as relics, invariant between tRNAs corresponding to different AAs in different species [20–23]. The calculated ring AL would be a good candidate of plausible primitive tRNA (Figure 2), and along with the other similar circular RNAs found through stability criteria, it might have been the raw material for the first biological systems.

## 3. RNA Relics

### 3.1. tRNAs Loops

From the well known Lewin’s archetypal tRNA [24], statistically obtained from all the known tRNA structures, we can deduce the common secondary structure (Figure 2, top left) showing a great interspecific invariance on levels of articulation, D, anticodon and T loops. Corresponding sequences observed for the Gly-tRNA of *Oenothera lamarckiana* or *Arabidopsis thaliana* [25–27] brought together match with AB and AL rings and hairpins having the same succession of critical triplets GGU and UCA whose pairing causes the tertiary tRNA structure (Figure 2, bottom).

### 3.2. Small RNAs

**Figure 3.**
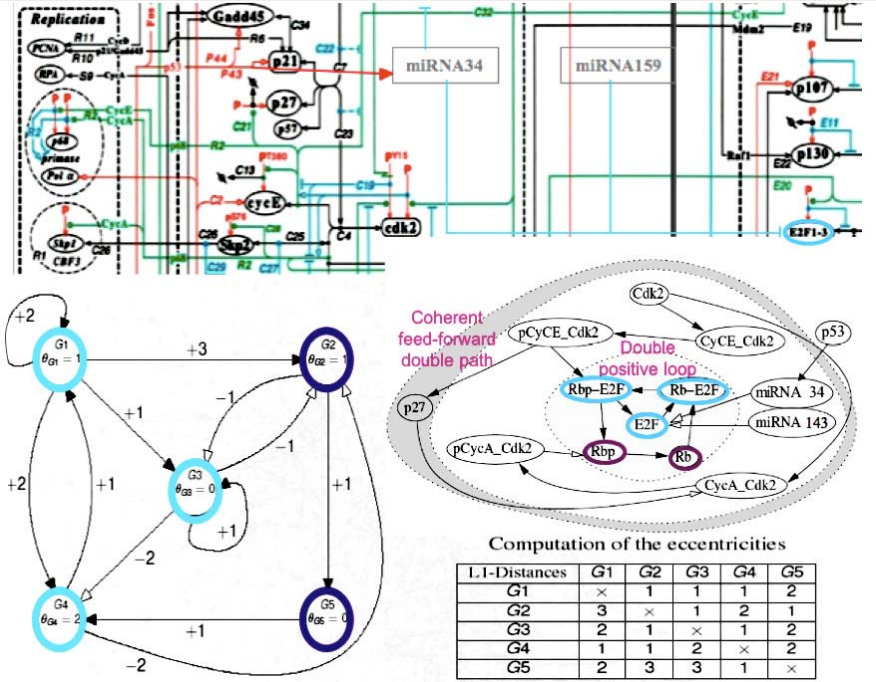
Schematic view of the cell cycle regulatory network (top, after [28]); zoom on the subnetwork centred on E2F (middle right); complexification of the subnetwork (middle left); computation of its eccentricities (bottom right). These representations highlight how miRs control globally the cell cycle by the manner they are connected to its regulatory network. Note the high eccentricity of miRNAs, revealing their upstream location in the regulatory system.

The small RNAs are made essentially of miRs and snRNAs from UTR genomes, and siRNAs. Their main action is a post-transcriptional control exerted essentially on the mRNAs, particularly for miRs, with the help of small proteins called RNA-binding oligo-peptides [28] within protein complexes like RISC. They belong to the frontier (in the graph sense) of the genetic regulatory networks, acting nearly as “Garden of Eden” on the central genes [29–32]. Their transcription can nevertheless be regulated by proteins expressed by these central genes like p53 [4]. It is for example the case of the regulatory network controlling the cell cycle [33] (Figure 3).

**Figure 4.**
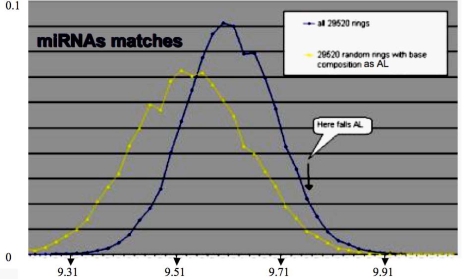
Distribution of the match values between the human miRs and the 29,520 rings solution of the variational problem (blue), and between these miRs and 29,520 random miRs with same base composition as the solutions.

By looking at the central subnetwork of the cell cycle control network, we can easily understand that the miRs 34 and 143 are on the direct boundary of a core made of the node having the lowest eccentricity (which is for a node the maximal length of the shortest pathways between this node and all other nodes). For example, the core in Figure 3, middle left, is represented by the three blue nodes G1, G3 and G4 of eccentricity 2, surrounded by two violet nodes G2 and G5 of eccentricity 3 (see the Table of eccentricities Figure 3, bottom left) realizing a double positive loop under the control of the miRs 34 and 43 having both eccentricity 5, and to the global boundary node p53 of eccentricity 6. Let us notice that the heads of the two coherent feed-forward double paths are Cdk2 and pCyCE_Cdk2 (see Figure 3, middle right) have an eccentricity equal to 5 and 4, respectively. The existence of attractors for the network dynamics is highly dependent on the boundaries, *e.g.,* fixing to a sufficient level p53 causes the occurrence of limit cycles in the parallel mode of updating the nodes [31,34]. The functional role of small RNAs is then critical for the cell metabolism. Because they are often very similar from one species to another, we compared their sequences (of mean length equal to 22 bases for the human miRs) to those of the archetypal loop AL, which is close to the Lewin’s tRNA loops. We see in Figure 4 that the match value (calculated as the mean number of their common bases) between the human miRs (from//mirdb.org/miRDB/) and the 29,520 ring solutions of the variational problem of Section 2, is better than for 29,520 random rings with same base composition as AL, the match value with AL falling in the 5% tail part of this last distribution, therefore the similarity between miRs and AL, with a significance p=0.05, is not due to chance.

By comparing AL with two specific miRs involved in the control of the cell cycle (miRs 34 and 143), both share a sequence of length 7 with AL (event of probability equal to 10^−4^):

**Table:** 

Match value with AL: 11/22	5′_ACAACCAGCUAAGACACUGCCA_3′:hsa-miR-34 a UUCAAGAUGAAUGGUACUGCCA
Match value with AL: 9/22	5′_UGAGAUGAAGCACUGUAGCUCA_5′:hsa-miR-143 UUCAAGAUGAAUGGUACUGCCA

**Figure 5.**
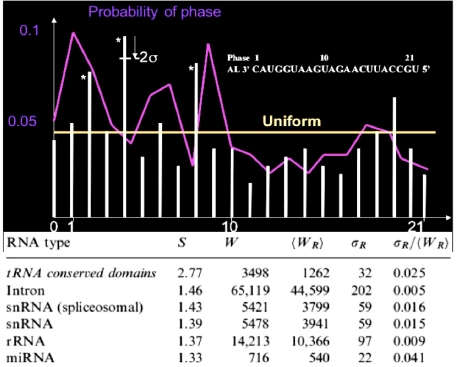
Phase histogram among the best matches between human miRs and AL (white bars) and between human miRs and the 29,520 rings (violet curve); number *W* of common substrings of length ≥ 5 between RNAs and AL, mean number <*W_R_*> and standard deviation <*W_R_*> for randomized rings, and the match score *S* = *W*/<*W_R_*> for small RNAs, tRNA conserved domains (loops), introns and ribosomal RNAs (rRNA) from the site www.sanger.ac.uk/Software/Rfam/.

When we calculate the matching phase between the human miRs and AL, then we observe significantly (p=0.001) three phases corresponding to the best matches, *i.e.,* the first base after the critical triplet GGU, the middle base of GGU and the base between the articulation A and GGU (Figure 5, top). This match is roughly respected by looking at the whole set of 29,520 variational solutions. The comparison between small RNAs and tRNA loops on one side, and AL on the other side done by counting their common substrings of length ≥ 5 (p=0.05), shows very significant similarities between RNA relics and this archetypal ring (Figure 5, bottom).

## 4. Similarities between Archetypal Genome, Viral Genomes and RNA Relics

**Figure 6.**
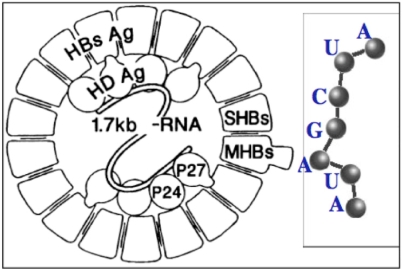
Circular Hepatitis D RNA (left) and miR substring of length 7.

By considering eight viruses with circular RNA of about 500 UTR bases (Figure 6) compared to about 500 human miR chains of mean length equal to 22 bases, we have observed 19,424 matches with substrings having more than 70% of similarity, involving 193,808 bases of which 143,394 are common, *i.e.,* a mean of 19,424/4,000 5 matches for each virus and miR, with a mean match value of 193,808/143,394 7.4, with 1/3 of perfect matches with substrings of length ≥ 7. The probability of such a perfect match is equal to: (¼)^7^+...+(¼)^22^ ≤ (Σ_i≥0_ (¼)^i^)/16,000 ≤ 1/12,000. With a probability of 1/12,000, we have a mean of 500x22/12,000≈0.9 for such perfect matches for one miR and one virus, hence a mean of 0.9x8x500 ≈ 3,600 ± 2x(4,000x0.9x0.1)^1/2^ ≈ 3,600 ± 40 for 500 miRs and eight viruses; it is significantly (p < 10–9) less than the observed number of such matches equal to about 6,000. Calculations for miRs 34 and 143 involved in the cell cycle regulatory network are given in Table 1.

**Figure 7.**
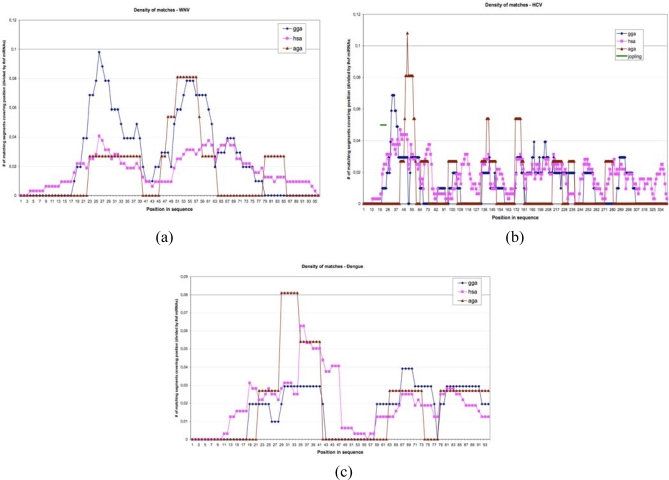
Matching curves between UTR viral (West Nile virus, hepacivirus and dengue), host (*Gallus gallus* and human) and vectors (*Aedes* and *Anopheles*) genomes.

The number of perfect matches between miRs 34 and 143 and the eight selected viruses is important and rare: a match value more than 10 has a probability equal to 0.01 and would occur only 0.1 ± 0.2 times for miR-143 and the eight viruses and not three times as in Table 1, which is a rare event having a probability of “large deviation” type to occur (<10^−6^). The best matches between human miRs and three viruses, West Nile (WNV), hepacivirus (HCV) and dengue, are given in Figure 7, showing that the percentage of segments of miRs matching the UTR parts of these virus fluctuates along the viral RNA, and that the integral of the matching curve is less important for a bird (Gallus gallus, gga) than for the human host and for the vector (hsa) in the case of non avian viruses (dengue and hepacivirus) and is more important for the vector Aedes or Anopheles (aga) involved in dengue and West Nile virus, but not in hepacivirus transmission [35].

On Table 2, we see 15 perfect matches of length ≥ 11, each of a probability of 0.00125, which would occur only 5 ± 2 × 2.232 times for the 500 human miRs and the eight viruses (p<10^−6^). All these observations are in favour of a long co-evolution of the virus, vector and host genomes with numerous exchanges explaining the present similarities.

## 5. Co-Evolution

**Figure 8.**
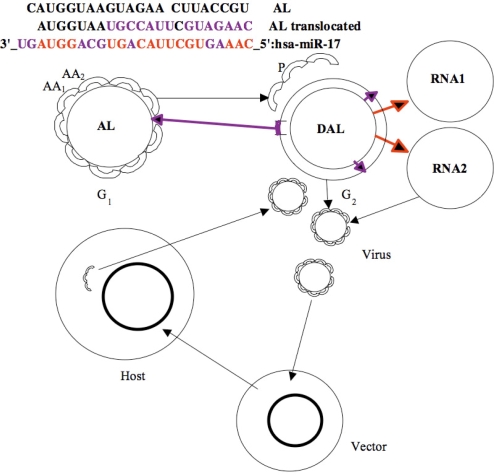
Evolutionary operators: Mutation/translocation/inversion/insertion (violet) and replication (red) on miR 17 (top); bi-directional exchange between AL RNA and its DNA version DAL as peptides P and RNA building machinery, and present host/vector/virus co-evolution process (bottom).

Our co-evolution hypothesis is based on the fact that on the one hand, the silencing machinery of hosts (its small RNAs) can serve to limit (or inhibit) viral attacks either by classical silencing on viral mRNA targets [10,11] or by plausible direct interactions between host and viral miRs [3], and, on the other hand, several viruses also have in their genome silencing systems that reduce (or inactivate) the immune response [36,37], and finally that this has forced a tight viral species to host species relationship leading to a co-evolution between the hosts and their viruses (Figure 8), probably often in a win-win configuration.

The win-win relationship between viruses and hosts is usually understood in the context of evolution over long times where viruses, even if they are dangerous for the considered species, act as genome editors and evolution accelerators [38,39]. However, numerous host-vector-virus systems are known in which quasi symbiotic associations exist, notably in hymenopter insects [40,41]. The same is true of the Epstein-Barr virus that infects 90% of the world human population and survives because of a huge, highly connected and very tolerant reservoir. In return, EB viruses confer immortality to human B lymphocytes.

RNA viruses have a strong propensity to mutate and a virus line can generate thousands of different copies within a single infected cell. We hypothesize that, compared to other viruses, this characteristic may confer them an additional ability to hijack the silencing systems of the host. MicroRNA based hijacking of viruses can occur i three different manners: (i) Viruses produce that way sequences less sensitive to the targeting action of the host cellular miRs. (ii) Moreover, generating thousands of variant copies of viral genomes within cells induces a huge variability of viral miRs, thus increasing the ability of viruses to silence the hosting systems. This assumption is supported by the fact that only one miR can silence hundreds of targets at the same time [5,6]. (iii) Finally, one must consider the dose effect: a population of numerous viruses targeted by the host silencing systems will saturate and stop them from playing their physiological role. Such an induced modification of the expression profile of the cell could lead to an enhancement of the viral development. Thereby, if some of the silencing mechanisms implied in the virus-host interactions are known, we think that only an approach based on the dynamics of populations of viruses and hosts can give a more realistic understanding of the viral infection and escape processes due to miRs. Those mechanisms are indeed often viewed in isolation and considered out of a dynamical context, but the ability of viruses to mutate during the infection, particularly on the sequences corresponding to the host cells miRs targets and the viral miRs, as well as their reproduction rates, are important criteria that have to be taken into account.

Nevertheless, a virus that would grow away from its fitness of interaction with its original host would risk to lose its ability to infect or on the contrary could destroy the pool of its target cells. This implies presumably that the selected viruses preserve a silencing machinery well adapted to their hosts – so with a weak variability – or that the hosts, forced by the evolution of their viruses, evolve by fitting their silencing systems to both their target viruses and their own physiological targets. A dynamical study of such viral ecosystems shall then be made in the context of evolutionary dynamics and percolation networks.

Hence we could simulate a genome able to mutate and then evolve under the constraint of having a phenotype adapted to its environment (physical, chemical, biochemical...) and the ability to reproduce itself alone (in the case of a cell) or by parasitising an autonomous genetic system (in the case of a virus). Some models were proposed [42] simulating in a very realistic manner the evolution of an artificial genome where genes are coded as sets of parameters that express a shape (a triangle). The composition of the expressed shapes in a geometrical space is the phenotype. For surviving to evolution (due to operations of punctual mutations, copies, insertions and deletions), the genome must express a phenotype close to an imposed fitness (global shape). The model however contains several limitations. It cannot simulate correctly its expression in the form of a “RNA milieu” and thus a proteome, hence it cannot represent the biochemical reactive relationships between their components. In that model, they result from a map of interactions between genes. It appears indeed difficult to realize a realistic functional representation, but several works have been done in that direction within the domain of typogenetics [43] or based on auto-catalytic sets of instructions and autopoietic automata such as Tierra [44], where the elements of a “digital reactive soup” are sets of functional instructions (in pseudo-code). Those sets are small programs that represent biochemical elementary actions such as “cut” or “copy” or “destroy”, coupled with probabilistic interactions [45,46]. These actions are analogue to the evolutionary operators “duplicate”, “insert”, “mutate”, “translocate”, “invert” or “delete” used in calculating the proximity between two RNAs like the shuffle similarity in [18,19], defined as the minimum number of cuts that need to be made in the second RNA sequence so that, after reordering the resulting pieces – with possibly deletion and inversion - we may obtain the first one (Figure 8).

Then, instead of talking about higher bids of attack and defence strategies, we shall talk about win-win strategies between hosts, vectors and viruses, that emerge from a “biochemical” dialogue and a reciprocal adaptation of their molecular systems (in particular the couples miRs-targets) according to a slow dynamics, on the time scale of species. An example exists, although its mechanism is not based on miRs and its time-scale is very short (shorter than the lifetime of an individual), that illustrates such a self-adaptive process between infected species (hosts) and infecting species (viruses, bacteria or parasites): it combines two processes, *i.e.,* on the one hand the lymphocyte clonal selection process for the Histo-compatibility Major Complex (HMC) and on the other hand the mutagenic process that confers to the infectious particles a certain resistance to the immune defence. During an infection, the antigen particles are presented by the macrophages to lymphocyte stem cells in the thymus. These cells are in a fast and important process of multiplication because of a strong stimulation by cytokines. Each one expresses a certain combination of HMC molecules, because of the huge number of possible arrangements of V(D)J genes [47–50]. Only few combinations are selected that correspond to the presented antigens. One talks about a clonal selection. A fast adaptation both of the immune system and of the infectious particles then occurs due to their mutability. Plasmid exchanges between bacteria constitutes another system of fast adaptation and resistance. Usually, these mechanisms are identified as acquired immunity because they are acquired during a short time, much smaller than the lifetime of an individual. On the contrary, we would tend to design the existence of miR links between viruses and hosts as an innate process. This is the case at the individual time scale because it is a slow process, but by considering now the species time scale, the notions of innate and acquired are not so clear. That way, another suggestion would be that miRs can represent the ancestral equivalent (with slow dynamics) of the evolved immune systems (functioning in comparison according to extremely fast dynamics). In that context, existing miRs (and probably also siRNAs) could be partially viewed as relics of an ancient biochemical immune system adapted to a world where the individuals were not much important because of the very high reproduction rates of the concerned species (bacteria or unicellular organisms), giving time for genetic adaptation to be fulfilled. Small RNAs have probably conserved this fundamental role but gained new functions in evolved organisms (existing cells), thus becoming integrated in their complexity.

## 6. Ancestral Processes Inherited from the Origins of Life

### 6.1. From the Discovery of the Genetic Code to the Birth of the Stereochemical Model

When the structure of the molecule of heredity was revealed by X-ray crystallography by R. Franklin and M. Wilkins [51,52] and then characterized as a double helix structure by J. Watson and F. Crick [53] in 1953, the scientific community had the possibility to search and find a molecular basis for explaining first the correspondence between DNA and proteins and then the now well-known processes of transcription and translation that allow protein production by the cell machinery [54–56]. G. Gamow, based on this new knowledge, proposed in 1954 his “diamond code” [57]. In his model, amino-acids can specifically dock into cavities formed by three nucleic bases of the two opposite strands of the DNA molecule, due to stereochemical preferences between acid, amine and radical groups with the nucleic bases. He was however not the first one to propose a stereochemical basis for the genetic code. This hypothesis is old and brings us back to the discovery of the DNA molecule itself (formerly called “nuclein”, a phosphored substance different to proteins and lipids) by F. Miescher, in 1869. At this time, the existence of proteins was known and F. Miescher was already thinking to the relationship between proteins and this new molecule in terms of stereochemistry.

In 1965 the correspondence between each amino-acid and one or several possible specific codons was finally discovered [58,59]. Since this time, research in molecular biology was mostly focused in how the translating machinery depends on the existence of adapter molecules, principally the tRNAs. The researches concerning the origins of life and the manner the first polynucleic assemblies, the XNAs (PNAs for peptide nucleic acids, RNAs or DNAs), coding for the first peptides or proteins followed a similar reasoning, *i.e.,* were also based on the principle of adapter molecules. These tRNAs ancestors would have been similar to the “aptamers” obtained in the *in vitro* evolution of amino-acids ligands [56,60–64]. Some chemists however have continued to work on a direct stereochemical interaction between amino-acids and DNA, for some of them because tRNAs were not yet or just discovered and later, for the others, because they thought aptamers or other adapters are too complicated for explaining DNA translation at the early stages of “life” and especially before, during the prebiotic ages [54,65–68]. Such a process occurring due to the presence of a sequence of XNA containing a succession of several codons in an amino-acids soup would favor the sequential and spatial getting-closer (an old peptidic “matrimonial agency”) of the corresponding amino-acids and would have a better chance to form peptides.

### 6.2. Models for the Emergence of a Genetic Code

This idea is one of the key pieces of the never-ending puzzle of how at least prebiotic lifelike forms appeared, and of the underlying question of how two complicated supra-molecular systems could appear at the same time and co-evolve until the emergence of basic forms of life [48]. Many hypotheses, all probably valid (there are certainly many origins to life), were proposed to try to explain the genesis of a primordial “genetic” code, *i.e.,* the way to maintain information in a durable molecule (*e.g.,* DNA) and exploit it by functional molecules such as proteins or more simple peptides having a catalytic activity. Two extreme hypotheses are the self-replication of XNA molecules (notably RNA) with no need for a peptide part, or the self-formation of peptides without XNA “catalysts” [55], but others are more intermediate such as a structural encoding in the form of a possible complementarity between secondary structures of peptides (such as α-helices) and repeated sequences of XNA nucleic bases [54]. The latter also supports the stereo-chemical scenario that allows the co-emergence of a triplet code and protein synthesis.

In prebiotic conditions (at least for *in vitro* conditions that aim to mimic them, such as in the famous Miller experiments) RNA and DNA polymers are not easy to form from single nucleotides (the synthesis of nucleotides is also very difficult under such reaction conditions) whereas a majority of amino-acids and some small peptides can form [69]. Peptides possibly appeared before XNAs, but rapidly a duality “information encoding” ↔ “functional catalytic systems” should have emerged, probably from a stereochemical coupling between XNAs (likely PNAs first, since they are easier to form because of their skeleton made of amino-acids, compared to the (desoxy)ribose one of RNA (resp. DNA)) and peptide structures probably associated in heterogeneous complexes composed of several simple chemical components (modules) that once assembled, even transitorily, form catalytic structures (or functional composomes [70,71]). Such structures (or thermodynamical minima) could be obtained in dynamic combinational chemistry by using sets of chemical compounds in a library [72]. Here we stress that not only stereochemical direct interactions between amino-acids and their respective current codons can constitute a primordial genetic code, but also that if some amino-acids have polymerized, they could in return help to confine nucleic bases so that their probability to polymerize into an XNA strand is increased. In addition to the coupled stereochemical and structural encoding [54], this process constitutes a good molecular basis for supporting theoretical works of artificial genetics such as typogenetics [43] that lead to the emergence and preservation of auto-catalytic loops from a dual system composed of XNA sequences (initially random sequences) and after translation (hypothesized) the corresponding set of peptides, some of which could have had a certain catalytic activity.

### 6.3. Experimentally Testing the Stereochemical Model for the Origin of the Genetic Code

Surprisingly, to our knowledge, the hypothesis of a stereo-chemically based primordial genetic code has been poorly tested experimentally in its original formulation. It has however been tested for aptamers, but the result was not very conclusive since it seems to work only for a few aptamers– amino-acid couples. As a research agenda, we propose here a possible experiment to test it *in vitro*. The objective of such an experiment would be double, that is to verify (i) that amino-acids “prefer” their corresponding nucleotide triplets, *i.e.,* their affinity is better for the latter than for others codons, and (ii) that two amino-acids (identical or different) can be located together on the sequence of their two adjacent corresponding codons (codon 1 and codon 2). The difficulty comes from the very weak expected interactions. So as to limit the influence of other competitive interactions, no cross or self-interactions must be allowed between hexanucleotides. Moreover, the sequences should not contain codons in the shifted frame that would code one of the amino-acids corresponding to codons 1 and 2 too; for example, the valid sequences we have selected are: (i) 3′-TCCCTC-5′ which corresponds to the Serine-Leucine dipeptide whose codons in the shifted frame are Proline codons CCC and CCT, or (ii) 3′=TCCTTC-5′ which corresponds to the Serine-Phenylalanine dipeptide, whose codons in the shifted frame are Proline CCT and Leucine CTT codons, or (iii) 3′-TGGTTT-5′ which corresponds to the Tryptophan-Phenylalanine dipeptide whose codons in the shifted frame are Glycine GGT and Valine GTT codons. Given the very weak expected levels of interaction, affinity chromatography techniques are not well-suited, but it is possible to measure the small chemical equilibrium shifts that should occur due to the weak association between specific amino-acids and their codons. Non specific interactions between codons and non-corresponding amino-acids should not generate such chemical equilibrium shifts. A good manner to study them is a dialysis using a membrane that lets amino-acids cross, but not hexanucleotides (membrane cutoff close to 1 to 1.5 kDa). The membrane would separate two containers containing two different solutions: (A) one with the nucleotides and a concentration C_t=0,A_ of amino-acids and (B) the other without nucleotides and with the same concentration C_t=0,B_ of amino-acids: C_t=0,A_ = C_t=0,B_ = C_t=0_. If the amino-acids associate, even weakly, with the hexanucleotides, then the concentration C_free_ of free amino-acids in this part of the container will decrease and, by dialysis, a new equilibrium between the concentrations of free amino-acids will take place progressively so as at the equilibrium C_t,A_ = C_t,B_ = C_free_. The non-free (temporarily associated) amount of amino-acids is such as C_associated_ = 2C_0_−(C_t,A_+C_t,B_) = 2(C_0_−C_free_). By measuring the concentration of amino-acids in the part B of the container at t=0 (C_0,B_) and after a long time (C_t,B_), we will be able to determine the affinity of specific amino-acids for their codons. The dosage of amino-acids will be obtained by fluorescent derivatization (for example by using FMOC chloride or fluorescamine) combined to chromatography separating techniques (HPLC) with a detection limit of about 10^−11^ mol.l^−1^. Another manner to measure this chemical equilibrium shift is by way of thermodynamic techniques, notably calorimetric micro-titration (ITC).

### 6.4. Implication of the Stereochemical Model in the Existing Living Systems

Further than these considerations on the origin of life, the existence of a reasonable level of direct interactions between amino-acids and their codons (even if it is infinitely lower than those mediated by tRNAs) should then not be ignored when talking about cell regulation, especially when their concentration gets high. Cell regulation is viewed at the gene level as a network implying transcription factors, produced by the products (proteins) of other genes. Cell regulation also occurs at the level of the translation due notably to the silencing action of miRs, or by way of post-translation modifications like phosphorylations. Finally, the ultimate layer of regulation is the functional one and is due to cell signalization, addressing, regulation of metabolic pathways by enzymatic activity, and regulation of energy levels (ATP, GTP amounts).

**Figure 9.**
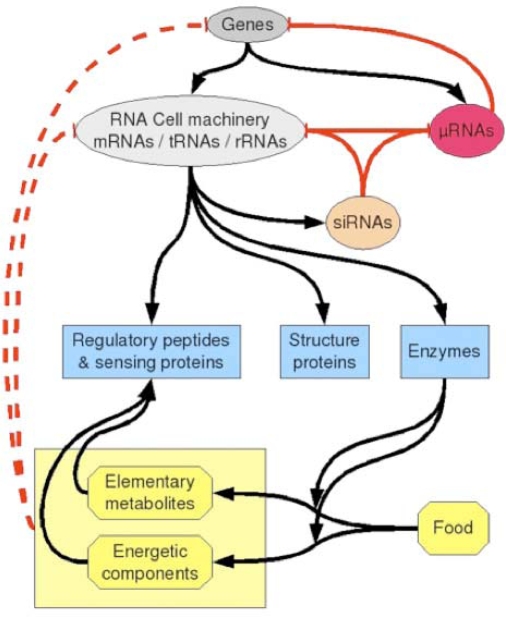
Schematic view of regulatory processes that can occur in a cell. Positive and negative classical regulations as well as syntheses are shown as black arrows. Negative regulations due to miRs and siRNAs are indicated as red arrows. A possible contextual down-regulation (dashed red lines) could come from direct interaction between metabolites (amino-acids and their derivatives, or energetic nucleotides) and RNA or DNA sequences.

Usually, the feedback action of the metabolism on the activity of the genes is only understood to be mediated by “sensors” (protein receptors) of particular metabolites. Those sensing molecules transmit the metabolic state of the cell to the genes and up or down regulate them. Another source of “global” regulation is the RNA interference (siRNAs) combined to the miRs and RNA-binding oligo-peptides activity [28]. It is due to the random garbage production of small sequences of RNA from the destruction of functional RNAs (mRNAs, tRNAs ...). Usually it is not really considered as a regulatory process and then called interference, but one can consider that even if these products are random, they apply a global down regulation to the cell machinery, so the living organisms had to develop (during evolution) robust and very efficient systems to survive despite of the presence of RNA interference. This natural process acts as a filter of efficiency of cell machinery subsystems. Direct interactions between metabolites (notably amino-acids) and RNA or DNA could be also viewed as interference, but we suggest that it shall be viewed as a context-dependent regulatory process. The internal levels of metabolites of an organism will depend on what it “eats”. When cells are cultured in media rich in certain metabolites, they adapt their metabolic pathways to use such metabolites, but in the same time, according to the stereo-chemical direct interactions, the same metabolites will exert a negative feedback on some cell functions because they will act as competitors with the tRNAs. What is amazing is that this regulatory process is specific since the targets concerned are the codons that stereo-chemically fit with the amino-acids: for example, if the medium becomes rich in Tyrosine and independently of its internal cell concentration, a negative feedback will appear that will inhibit the formation of all proteins that contain Tyrosine residues. Of course, such an inhibition should be very weak, but it could act as a bias on cell activity. At a rougher level, the same can be said with single nucleotides such as energetic nucleotides (ATP, GTP), that could slow down the formation of proteins whose mRNAs are rich in complementarity bases (resp. Uracil and Cytosine). Such a situation could occur when cells are in particular energy states. In Figure 9, a schematic view of cell regulation is proposed, showing in dashed red lines the negative feedback the metabolism could exert on the cell machinery and on the genome.

## 7. Conclusions

We have presented some arguments based on similarities between RNA relics (notably tRNAs loops and small RNAs) and both viral and archetypal genomes, in favour of a co-evolution of still evolving genomes (early the archetypal and now the viral one’s), due to environmental influences. This co-evolution and the presence of ancestral mechanisms such as the relics of a plausible biochemical immune system or a stereochemical direct interaction between metabolites and XNA sequences coming from the origins of life, allowed the constant enrichment of the genome of the more recent species as well as the increase of their adaptive ability to survive in an infectious environment both source of positive evolutionary and negative regression processes. Concerning the negative repression, human miRs are indeed clearly involved in cancer progression [73–77], in particular at the level of cell proliferation; for example, miR-34 and miR-143 are elevated in lung cancer [76]. Non-genetic heterogeneities coming from the presence of multiple attractors due to these miRs expression [31,34] are then invoked as mutation-independent driving forces for the somatic evolution of tumours [77]. Concerning the positive evolution, the miRs genes and their target sites are under Darwinian selection and continue to evolve increasing the corresponding species fitness [78,79], which gives to RNA relics a central role at the origin and in the evolution of life.

## Figures and Tables

**Table 1.** Perfect matches ≥ 7 (green) between miR-34, miR-143 and eight viruses.

**miR**	**Virus**	**Number**	**Extr**	**Match**	**start**	**end**	**start**	**end**	**Aligned sequence**
miR-34	Jap. encephalitis	-	3−	8	3	10	184	177	aatcagct
miR-34	Jap. encephalitis	rAT	3+	8	4	11	522	529	atcagcta
miR-34	Jap. encephalitis	-	3−	7	9	15	19	13	ctaacta
miR-34	Yellow fever	-	3−	8	16	23	234	227	cactgcct
miR-34	Pestivirus	-	3+	7	6	12	101	107	cagctaa
miR-34	Pestivirus	-	3−	7	6	12	170	164	cagctaa
miR-34	Pestivirus	-	3+	8	12	19	145	152	actacact
miR-34	Pestivirus	-	3−	7	6	12	174	168	cagctaa
miR-34	Hepacivirus	H77-pH21	5+	7	17	23	288	294	actgcct
miR-34	Hepacivirus	1b	5+	7	17	23	288	294	actgcct
miR-34	Hepacivirus	JHF-1	5+	7	17	23	287	293	actgcct
miR-34	Hepacivirus	JHF-1	3+	8	7	14	27	34	agctaact
miR-34	Uncl. Hepacivirus	JPUT971017	5+	7	17	23	288	294	actgcct
miR-34	Uncl. Hepacivirus	JPUT971017	3+	7	13	19	16	22	ctacact
miR-34	Hepacivirus	3a	5+	7	17	23	286	292	actgcct
miR-34	Flaviviridae	b	5+	7	17	23	392	398	actgcct
miR-34	Flaviviridae	c	3+	7	16	22	165	171	cactgcc
miR-34	Phlebovirus	segM	3+	7	7	13	43	49	agctaac
miR-34	Phlebovirus	segM	3−	7	6	12	230	224	cagctaa
miR-34	Hepatovirus	-	5−	7	15	21	353	347	acactgc
miR-143	Dengue	2	3−	10	1	10	147	138	tgagctacag
miR-143	Dengue	2	3−	11	12	22	280	270	gcttcatctca
miR-143	Dengue	3	3−	7	8	14	273	267	cagtgct
miR-143	Dengue	4	3−	9	2	10	82	74	gagctacag
miR-143	Jap. encephalitis	-	5+	7	14	20	6	12	ttcatct
miR-143	Jap. encephalitis	-	3−	10	12	21	252	243	gcttcatctc
miR-143	Jap. encephalitis	rAT	3−	7	3	9	558	552	agctaca
miR-143	Jap. encephalitis	-	5+	7	7	13	63	69	acagtgc
miR-143	Jap. encephalitis	-	3−	7	3	9	248	242	agctaca
miR-143	Jap. encephalitis	-	3+	8	4	13	305	314	gctacagtgc (miR) gcgacagtgc (virus)
miR-143	Jap. encephalitis	-	3−	8	1	10	462	453	tgagctacag (miR) tgacctacag (virus)
miR-143	Jap. encephalitis	-	3−	7	3	17	51	37	agctacagtgcttca (miR) agctaaacttctaca (virus)
miR-143	Tick-borne enc.	w	3+	7	1	7	236	242	tgagcta
miR-143	Tick-borne enc.	w	3+	8	11	18	262	269	tgcttcat
miR-143	Tick-borne enc.	w	3−	7	10	16	590	584	gtgcttc
miR-143	Tick-borne enc.	w	3−	8	11	18	379	372	tgcttcat
miR-143	Pestivirus	-	5−	7	6	12	353	347	tacagtg
miR-143	Hepacivirus	JPUT	3+	7	3	9	14	20	agctaca
miR-143	Uncl. Flaviviridae	a	5+	7	4	10	444	450	gctacag
miR-143	Uncl. Flaviviridae	b	5−	9	5	13	268	260	ctacagtgc
miR-143	Uncl. Flaviviridae	c	3+	7	7	13	248	254	acagtgc
miR-143	Uncl. Flaviviridae	c	3+	7	14	20	7	13	ttcatct
miR-143	Uncl. Flaviviridae	c	3−	7	7	13	259	253	acagtgc
miR-143	Phlebovirus	segM	3+	7	9	15	180	186	agtgctt
miR-143	Phlebovirus	segS	5+	7	9	15	16	22	agtgctt

**Table 2.** Some best perfect matches ≥ 11 between human miRs and 8 viruses.

**miR**	**Virus**	**Number**	**Extr**	**Match**	**start**	**end**	**start**	**end**	**Aligned sequence**
miR-21	Jap. encephalitis	-	3+	11	2	12	549	559	caacatcagtc
miR-422	Uncl. Flaviviridae	c	5+	12	9	20	420	431	gactccaagtcc
miR-490	Yellow fever	-	3+	11	12	22	252	262	cctccaggttg
miR-495	Tick-borne enc.	w	3−	11	8	18	565	555	tgcaccatgtt
miR-500	Uncl. Flaviviridae	c	5−	11	9	19	315	305	ttgcccaggtg
miR-511	Hepacivirus	H77-pH21	3+	11	1	11	180	190	tgactgcagag
miR-511	Hepacivirus	1b	3+	11	1	11	190	200	tgactgcagag
miR-511	Hepacivirus	JHF-1	3+	11	1	11	194	204	tgactgcagag
miR-511	Hepacivirus	JPUT9710	3+	11	1	11	169	179	tgactgcagag
miR-520	Dengue	4	3+	11	5	15	15	25	caccaaagaga
miR-9	Pestivirus	-	3−	11	1	11	175	165	tcatacagcta
miR-93	Pestivirus	-	3+	11	12	22	159	169	aacagcacttt
Let-7	Pestivirus	csfv	3+	11	4	14	121	131	gtacaaactac
miR-143	Dengue	2	3−	11	12	22	280	270	gcttcatctca
miR-453	Pestivirus	bvdb	5−	12	1	12	160	149	cgaactcaccac
